# Weather and biotic interactions as determinants of seasonal shifts in abundance measured through nest-box occupancy in the Siberian flying squirrel

**DOI:** 10.1038/s41598-020-71391-2

**Published:** 2020-09-02

**Authors:** Vesa Selonen, Kari Hongisto, Mikko Hänninen, Tytti Turkia, Erkki Korpimäki

**Affiliations:** 1 Section of Ecology, Department Biology, University of Turku, 20014 Turku, Finland; 2 Tieteenkatu 6 A 66, 33720 Tampere, Finland; 3 Varpulantie 490, 62220 Pernaa, Finland

**Keywords:** Population dynamics, Ecology, Forest ecology, Behavioural ecology

## Abstract

It is much debated whether the direct effects of weather or biotic interactions determine species’ responses to climate change. For example, an important biotic factor for herbivores in northern ecosystems is the availability of winter food. If the food availability changes because of the changing climate, it likely has major impact on the abundance of herbivores. To evaluate this, we need to know the relative roles of weather and biotic interactions, such as food availability and risk of predation, for the species. Here, we utilize long-term data on nest-box occupancy by Siberian flying squirrels (*Pteromys volans*) in Finland during 2002–2018. We built binary models with nest-box occupancy in different seasons as a response variable. Weather, winter food (tree mast), and predator presence (the Ural owl, *Strix uralensis*) modified seasonal nest-box occupancy patterns of the flying squirrel. However, the effect of weather was only important in the summer. The negative effect of predators was clear for adults but, surprisingly, not for overwinter survival of apparent juveniles. Considering the relative importance of different factors, winter food availability had a clear positive effect in each season. Our study supports the view that the effects of climate change mediate through multiple biotic interactions. In forest ecosystems, responses of masting trees to weather likely play an important role in species responses to climate change.

## Introduction

The consequences of global warming for the range shifts of both flora and fauna are well demonstrated^1–3^. We need, however, better understanding on whether the climate affects species directly through the changes in local weather or through changes in biotic interactions^4–6^. The changes in weather, both in temperature and in precipitation, have varying effect on the different species in the community. Consequently, disturbed trophic interactions may threaten species that otherwise would be tolerant to changing climate^7,8^.

For herbivores in northern ecosystems, an important factor determining their responses to climate change probably relate to the response to weather by plants that provide winter food^9^. Winter temperature has been rapidly increasing in northern latitudes during the last decades, which is expected to cause clear changes in the ecosystems of these regions^10^. For example, increased temperature in winter and spring typically advances phenology of plants that affect breeding success of herbivores^11,12^. In addition, avian communities, and thus the abundance of avian predators, are undergoing changes in distribution in northern latitudes. Particularly, the northern species appear to decline due to climate change, whereas many forest specialists have suffered from extensive forest exploitation^13^. To understand how climate change will affect a species, the effects of weather should be analysed in relation to the effects of food resources and predation pressure^14,15^.

The responses to weather and biotic factors determining survival may also differ between individuals^12^. Overwinter survival is typically challenging for juveniles that may lack the experience and resources available for adults. For example, in arboreal squirrels, the overwinter survival of juveniles is generally low, partly due to juveniles’ poor ability to locate high-quality territories or food patches^16,17^, and also due to the inherent boom-bust dynamics of the resource-pulse system in which they live^18,19^. Tree mast forms a resource pulse with years of abundant forage and years when food is scarce for arboreal squirrels. Furthermore, the main habitat of squirrels, boreal forest, is often under heavy human use, which makes them exposed to habitat loss and degradation^20^. Thus, predicting how these species will respond to climate change depends also on other factors than only the direct effects of weather on individual performance.

We studied the nest-box occupancy as mean to determine abundance of an arboreal squirrel living in northern latitudes, the Siberian flying squirrel (*Pteromys volans*, hereafter the flying squirrel). The flying squirrel is a nocturnal sciurid that lives in mature boreal spruce-dominated mixed forests, nesting in cavities, nest boxes and twig dreys, and feeding on leaves, catkins, seeds, and buds of deciduous trees^21^. The species prefers edge habitat to some extent and can live in small forest fragments^22,23^, but is a forest specialist that suffers from habitat loss and degradation in a managed forest landscape^21,24^. The winter food availability (catkin mast of deciduous trees) determines reproductive success in flying squirrels by advancing spring reproduction and allowing production of second litters in summer^22,25^. Increased precipitation in winter is also positively correlated with both the reproductive success^25^ and the population growth rate^26^, possibly due to increased spring foliage growth in moist conditions. The nocturnal Ural owl (*Strix uralensis*) is the main predator of the night-active flying squirrel, and they have been shown to detrimentally impact the occurrence of flying squirrels in the landscape^23,27^. However, the knowledge on relative roles of effects of habitat, food, weather, and predation in different life stages of the flying squirrel are lacking.

**Figure 1:**
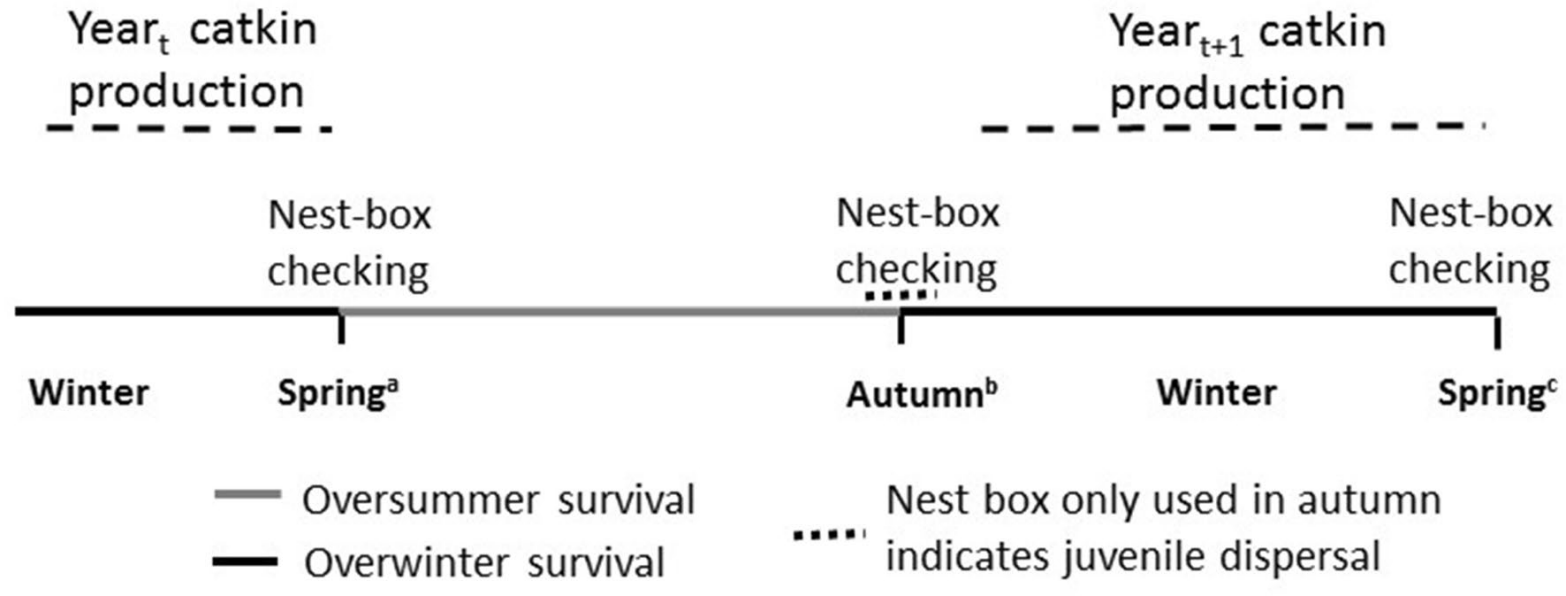
Timeline of data collection for flying squirrel nest-box occupancy and for the period when catkins of deciduous trees are consumable for flying squirrels (dashed line). Catkin availability in year_t_ is expected to increase juvenile production and in year_t+1_ to affect winter survival. ^a^In spring, nest boxes can be used only by resident adults (1 year or older); ^b^in autumn, nest boxes can be used by dispersing juveniles and resident adults: a nest box used in spring, but not in autumn indicates adult summer mortality. A nest box not used in spring, but used in autumn indicates juvenile dispersal. ^c^Occupancy of a nest box after winter indicates overwinter survival of an individual occupying the nest box in the previous year.

We investigate winter food availability, season-specific effects of weather and predation pressure on nest-box occupancy of flying squirrels. We use long-term data on nest-box occupancy in spring, autumn and the following year (spring_t+1_; Fig. 1) collected in western Finland. Based on mortality and dispersal patterns we have identified three patterns in nest-box occupancy from the records. The dispersal of the flying squirrels occurs in autumn, when juveniles born in spring leave their mothers home range (breeding dispersal is rare in flying squirrels^28,29^). Therefore, (i) a nest box not used during the breeding season in spring but used after the breeding season in autumn likely reflects the arrival of dispersing juveniles to the site (hereafter *dispersal model*). (ii) A nest box occupied in spring but not the following autumn probably reflects the mortality of adult residents during summer (flying squirrels are partly territorial^29^; hereafter *summer survival model*). Finally, (iii) nest-box occupancy in the following year (spring_t+1_) reflects overwinter survival (hereafter *winter survival model*).

Our main aim is to evaluate the possible pathways of how climate change may affect the flying squirrel: does weather affect the species directly, or does the effect of climate change more likely mediate through changes in food availability or predation risk? We predict that the effects of weather, winter food availability and predator pressure will vary for the seasonal occupancy models (i–iii above). For dispersal model (i), we predict that the availability of winter food (alder catkins, *Alnus* sp.) in previous winter increases offspring production^22,25^, which is reflected in an increased numbers of cases when nest boxes are occupied only in autumn due to immigration. Birch (*Betula* sp.) catkins are important winter food, but they are of lesser importance for reproduction than alder catkins^22,25^. Increased precipitation in winter and spring also may enhance survival of juveniles (see^25^) and thus increase the number of dispersers. For summer survival model (ii), presence of the Ural owl^23^ should decrease and moist summer weather might increase oversummer survival of adults, because drought (interaction of warm weather and little precipitation) likely has adverse effects on food availability for flying squirrels in summer. Warm summer temperature may be advantageous during summer, but it also increase catkin availability in next winter^26,30^. For winter survival model (iii), we predict that warm winter temperature and high birch catkin availability increase overwinter survival. The presence of the Ural owl should decrease the overwinter survival in sites occupied by natal dispersers more than that of resident adults. Similar interaction can be predicted for the effect of winter food availability, that is, the lack of food is assumed to affect juveniles (boxes occupied only in autumn) more than adults (boxes occupied whole summer).

## Results

Altogether, the data included 757 observations of a flying squirrel occupying a nest box (Table 1). In 25% of these the nest box was occupied only in spring (n = 191), in 50% (n = 376) both in spring and autumn, and in the remaining 25% only in autumn (n = 190; the possible natal dispersal cases).

**Table 1:** Data for yearly nest-box occupancy and values for weather and winter food data used in this study.

Occupied nest boxes						Food and weather variables								
Year	Spring only	Spring and autumn	Autumn only	Alder pollen		Birch catkins	SpringT	SpringR	SummerT	SummerR	AutumnT	AutumnR	WinterT	WinterR
2002	4	7	3	1,680		359	7.9	27	16.9	74	0.7	27	− 5.4	30
2003	12	1	2	700		310	5.6	40	15.5	66	4.3	47	− 7.6	20
2004	3	7	11	1,800		82	7.1	38	14.4	63	4.2	72	− 4.7	24
2005	6	17	10	5,104		231	6.3	21	15.6	86	6.6	48	− 4.4	27
2006	11	12	10	1,520		538	6.6	49	16.5	19	5.8	65	− 7.6	20
2007	32	12	5	253		435	6.7	34	15.3	67	4.5	36	− 4.1	45
2008	15	26	9	650		287	6.5	20	13.8	81	4.5	44	− 1.4	42
2009	19	20	7	1,270		201	7.0	37	13.4	51	4.6	30	− 4.5	30
2010	18	23	18	2,400		328	11.0	45	17.3	80	2.8	47	− 9.6	32
2011	19	33	13	500		62	7.3	30	16.5	85	6.9	59	− 9.5	30
2012	4	47	24	570		1,243	5.3	58	14.4	83	4.9	69	− 4.3	33
2013	16	43	13	300		31	7.5	27	15.6	80	5.5	46	− 7.3	34
2014	6	41	19	3,000		892	7.0	30	15.7	80	4.9	47	− 2.1	30
2015	14	41	12	490		130	6.4	60	14.1	64	6.2	56	− 1.8	27
2016	4	30	18	970		44	7.6	29	14.9	107	3.9	33	− 4.0	36
2017	8	16	16	1,750		70	3.8	32	13.8	57	4.9	43	− 3.1	25
2018	NA^a^	NA	NA	290		351	NA	NA	NA	NA	NA	NA	− 6.3	24
x̄ ± SD	15 ± 9	23 ± 14	12 ± 6	1,367 ± 1,249		329 ± 320	6.9 ± 1.5	36 ± 12	15.2 ± 1.2	71 ± 19	4.7 ± 1.5	48 ± 13	− 5.2 ± 2.5	30 ± 6.8

Total number of nest boxes occupied by flying squirrels in spring only, both in spring and in autumn, and only in autumn. Alder pollen as pollen in 1 m^3^ of air, birch catkins as catkins per tree; temperature as °C; and rain as mm/month.

*T* temperature, *R* rain, *NA* data not available.

^a^For 2018, we had only data for spring occupancy (18 sites from year 2017 were occupied in spring 2018).

**Figure 2:**
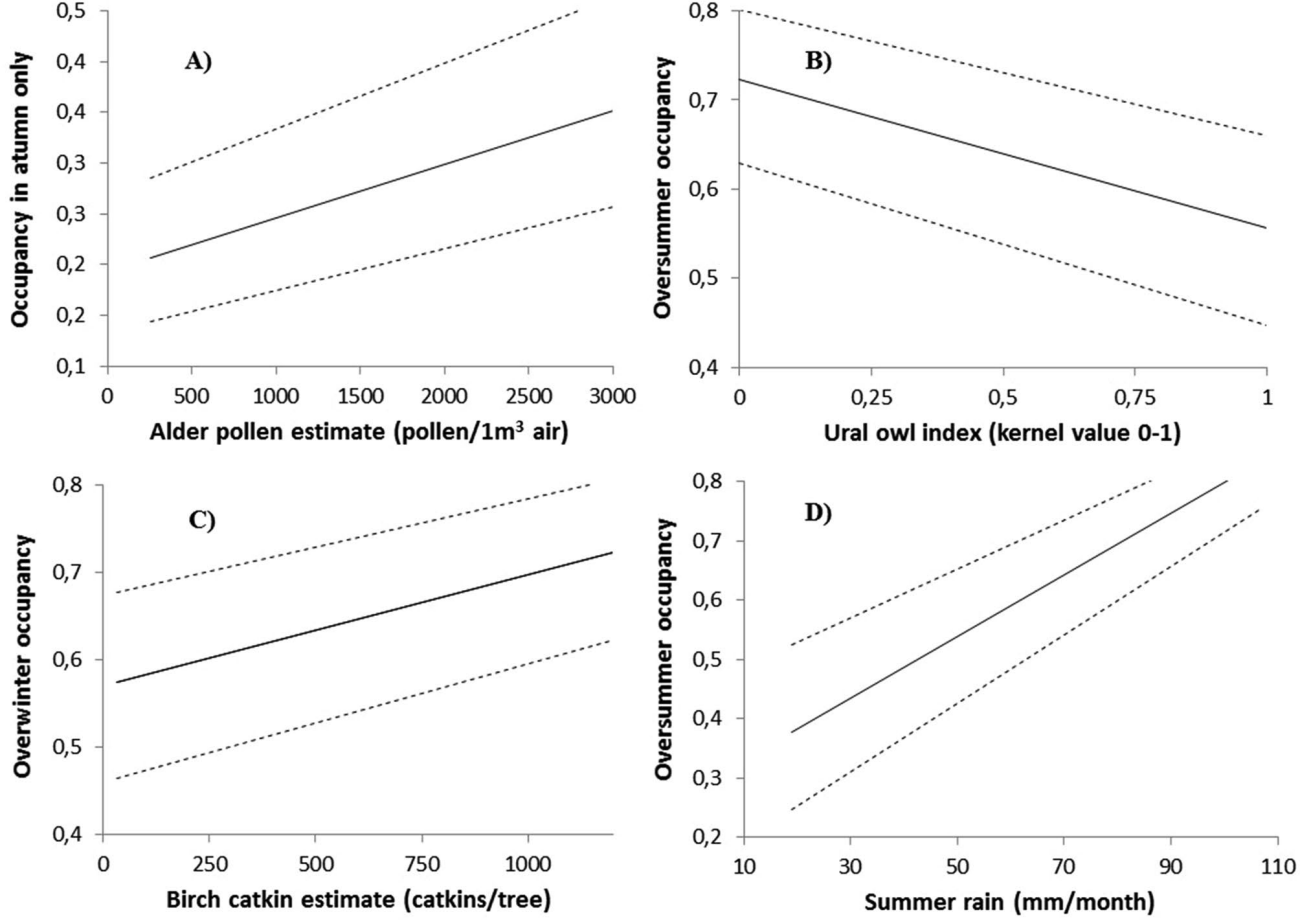
Predicted effects of environmental variables on flying squirrels during 2002–2018 based on binary models of nest-box occupancy in different seasons. (**A**) Occupancy of nest boxes used only in autumn (potentially occupied by dispersing juveniles) in relation to alder pollen in previous spring (index for winter catkin production); (**B**) oversummer survival, that is, occupancy of nest boxes from spring to autumn, in relation to Ural owl presence index; (**C**) overwinter survival in the nest-box use in relation to birch catkin estimate in winter; (**D**) oversummer survival in relation to precipitation in summer. Line based on predicted mean values with upper and lower 95% confidence intervals (dashed lines).

*In the dispersal model (i)*, the likelihood for nest boxes become inhabited during summer (occupied in autumn) was positively related to the alder pollen estimate from the previous spring (Fig. 2a, Table 2). Weather variables had no effect on nest-box occupancy in autumn only (Table 2), but predator pressure (the Ural owl index) had a slight positive effect in the model.

**Table 2:** The effect of environmental variables on the likelihood for nest boxes to be used only in autumn (probable natal dispersal cases; the dispersal model) and for nest boxes to be occupied oversummer vs. only in spring (the summer survival model).

Model for nest-box use*	Estimate	Test measure: F_df_	p
**Autumn only vs spring only/oversummer (n = 757)**			
Alder pollen	0.26 ± 0.09	8.5_1,587_	**0.003**
Ural owl index	0.27 ± 0.12	4.2_1,587_	**0.01**
Summer rain	0.06 ± 0.11	0.3_1,587_	0.60
Summer temperature	− 0.05 ± 0.10	0.3_1,587_	0.61
No. of nest-box visits	0.02 ± 0.01	2.0_1,587_	0.15
Year	0.04 ± 0.11	0.1_1,587_	0.74
**Oversummer vs spring only (n = 567)**			
Birch catkins	0.35 ± 0.10	11.7_1,347_	**0.0007**
Ural owl index	− 0.44 ± 0.21	4.3_1,347_	**0.03**
Winter temperature	0.09 ± 0.10	0.9_1,347_	0.35
Summer rain	0.29 ± 0.09	11.6_1,347_	**0.0007**
No. of nest-box visits	0.02 ± 0.11	0.03_1,347_	0.86
Year	0.45 ± 0.13	11.4_1,347_	**0.0008**

Negative estimates indicate negative effect on occupancy in autumn only or oversummer.

p < 0.05 is in bold.

*Amount of habitat had no effect in either of the models (1. model: F_1,551_ = 0, p = 0.96; 2. model: F_1,330_ = 0.03, p = 0.87); Alder or birch and best fitted weather variables were selected to final models based on AIC comparison, see “Materials and methods”.

*In the summer survival model (ii)*, occupancy was negatively related to the Ural owl index (Fig. 2b), whereas birch catkin production from the previous winter and summer precipitation (Fig. 2d) had positive effects on nest-box occupancy (Table 2). Summer temperature or interaction between temperature and precipitation had no effect. The oversummer occupancy increased during the study period (2002–2018, Table 2).

**Figure 3:**
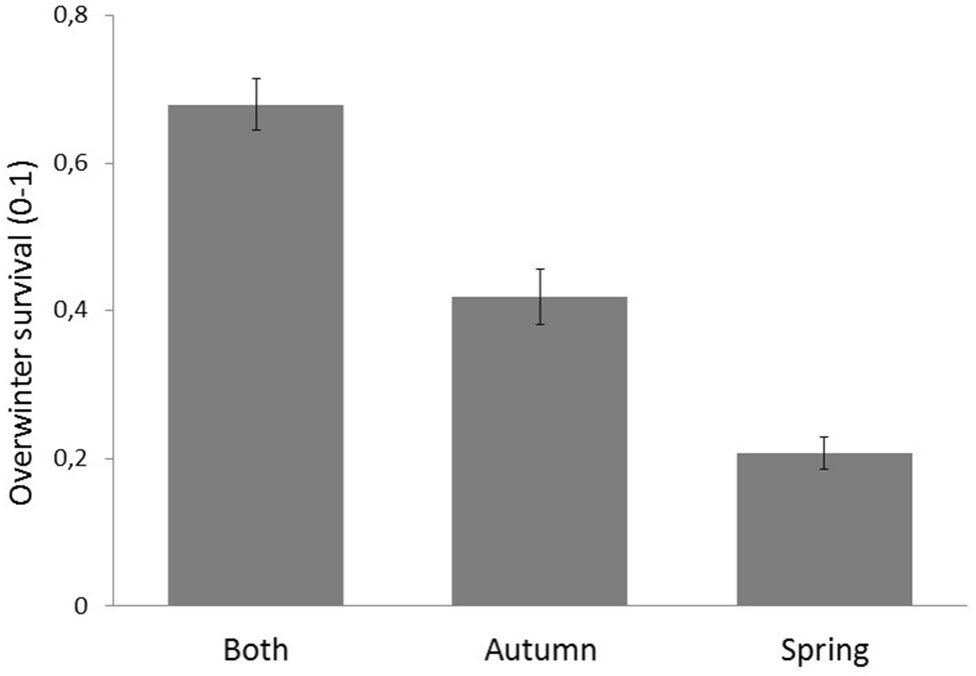
Predicted overwinter survival (mean values with upper and lower 95% confidence intervals) based on binary model for nest-box occupancy by flying squirrels. That is, the likelihood for a nest box to be occupied the following spring, after winter, for nest boxes occupied only in the previous spring (n = 191), both in the previous spring and autumn (n = 376) and in autumn only (n = 190; the possible dispersal cases).

*In the winter survival model (iii)*, occupancy after winter was positively related to food availability in winter (birch catkins, Fig. 2c) and to temperature in the preceding summer (the only weather variable linked to winter survival; Table 3). The overwinter occupancy decreased during the study period (Table 3) and was lower in nest boxes used only in autumn (dispersal boxes) compared to nest boxes used both in spring and autumn (resident boxes; Fig. 3). Interaction term between season (autumn/spring and autumn) and winter food availability was not statistically significant, but there was a significant interaction between season and Ural owl index (Table 3). The negative effect of Ural owl was clear for resident boxes (spring and autumn; estimate − 0.51 ± 0.20, F_1,263_ = 6.7, p = 0.01) but not for disperser boxes (autumn only; estimate − 0.07 ± 0.21, F_1,68_ = 0.1, p = 0.75; the same model as in Table 3, but separately for the seasons and without interaction terms). Amount of forest around the nest-box site had no effect in any of our models (Tables 2, 3).

**Table 3:** Effects of environmental variables and season on observed overwinter survival for flying squirrels during 2002–2018 (nest boxes occupied vs not occupied after winter, that is, the following spring.

Variable	Estimate	Test measure: F_df_	p
Season	SA: 0, A: − 0.64 ± 0.2	9.0_1,80_	**0.003**
Ural owl index	− 0.5 ± 0.2	2.8_1,417_	0.09
Season*Ural owl index	SA: 0, A: 0.49 ± 0.2	4.4_1,417_	**0.03**
Birch catkins	0.26 ± 0.15	6.4_1,417_	**0.01**
Season*Birch catkins	SA: 0, A: 0.11 ± 0.23,	0.2_1,417_	0.63
Summer temperature	0.22 ± 0.1	4.4_1,417_	**0.04**
Winter temperature	− 0.13 ± 0.12	1.0_1,417_	0.31
Autumn rain	− 0.05 ± 0.11	0.2_1,417_	0.66
No of nest-box visits	0.12 ± 0.15	0.7_1,417_	0.40
Winter rain	0.22 ± 0.12	3.4_1,417_	0.07
Year	− 0.32 ± 0.13	6.2_1,417_	**0.01**

*A* autumn-only occupancy, *SA* oversummer occupancy, i.e. both in spring and autumn; spring only boxes omitted form the data, n = 565). Binary mixed models with nest-box site as a repeated factor. Negative estimate values indicate lower overwinter survival.

p < 0.05 is in bold.

*Weather variables were selected based on AIC comparison, see methods; When we used alder instead of birch, alder had no effect (F_1,417_ = 0.4, p = 0.5); Adding amount of forest around the site to the model had no effect (F_1,332_ = 1.0, p = 0.2); modelled separately due to missing data, see “Materials and methods”.

## Discussion

In this study, weather, winter food, and Ural owl presence modified seasonal nest-box occupancy patterns of the flying squirrel. Considering the relative importance of different factors, winter food availability had a clear positive effect in each season following our predictions. The Ural owl presence had negative effect, but surprisingly did not affect the overwinter survival of apparent juveniles. For weather, the responses were less clear, but precipitation had clear positive effect on the oversummer nest-box occupancy of flying squirrels.

In contrast to recent avian studies, which have revealed the importance of direct effects of weather on the performance of individuals (e.g.^15,31,32^), our results emphasize the role of biotic interactions in the population dynamics of the species. For example, the winter weather had little or no direct effect on apparent overwinter survival in flying squirrels. In earlier studies on flying squirrels, increased winter precipitation increased the reproductive output^25,26^, and there is an optimum winter temperature that determines regional occurrence patterns of flying squirrels (^27^, see also^33^). However, these previously observed correlations between weather and the species might result from the indirect effects of weather on the food resources of flying squirrels, not the direct effect of weather on individuals^25,26^. Accordingly, in this study summer temperature was positively linked to apparent overwinter survival, which may be related to the fact that warm summers predict good catkin mast for the following winter^26^. This is possible, because our winter food indexes measured the variation in catkin mast at very coarse level.

From a conservation perspective, it is encouraging that winter weather appeared not to have negative effect on nest-box occupancy of flying squirrels, because overwinter survival determines population dynamics of vertebrates in northern latitudes to a large extent (e.g.,^34,35^). Instead, our results support the earlier studies indicating positive effects of precipitation on flying squirrels^26,36^, as precipitation in summer very clearly increased oversummer survival in our data. In the summer, the flying squirrel depends on plant material other than catkins (leaves, buds etc.,^21^), and the quality of the plant material is negatively affected by drought. However, temperature increases seemed to have no or positive effects on flying squirrels, which may indicate that the species is not directly threatened by rising temperatures in the near future. Similarly, in North American red squirrels (*Tamiasciurus hudsonicus*), warm spring temperatures are observed to decrease reproductive failures and thus increase female fitness^37^.

Winter food supply has a central role in the life of arboreal squirrels (e.g.^19,38,39^) and in our study it modified occupancy patterns during all the studied seasons. For example, in the North American red squirrel, which is highly dependent on stored food, the effect of food exceeds the effect of spring weather on reproduction^39^. In the flying squirrel, the number of apparent dispersers occupying free territories in the autumn and the apparent survival both in summer and winter, all were positively related with catkin availability in winter. Catkins flower in the beginning of the breeding season, but can be stored by flying squirrels to prolong the period during which the catkins can be consumed and increase reproductive output (^22,25^, but see ^26^, where the lack of effect of winter food may be related to the timing of censuses). It remains unknown how often and in what amounts flying squirrels makes these storages, perhaps because they are often located high up in spruce branches. Sometimes stores can be in cavities^21^, and, in our study area, the annual mean number of located catkin storages of flying squirrels was 2.2 (± 1.7). Of these, 33 were found in boxes set up for Boreal owls (*Aegolius funereus*) within our study area (see^40^), and only two in boxes for pygmy owls (the boxes used in the current analysis). The use of the former nest-box type (with a larger entrance-hole and bottom area than in the boxes used for nesting by flying squirrels) may indicate that flying squirrels do not prefer making storages in cavities used for nesting. All catkin storage observations were made during the spring.

The population dynamics of a species dependent on tree mast is expected to follow boom-bust dynamics, where high reproductive success after mast is followed by low juvenile survival the following winter, when food availability crashes^41,42^. However, against this prediction, the response to winter food availability in overwinter survival did not differ between nest boxes occupied by apparent yearlings (dispersers) and residents. In previous arboreal squirrel studies, it has been found that juveniles experience severe mortality that varies drastically between years, but those individuals that survive the first winter have a high probability of survival^18,19^. Overwinter survival of juveniles is low compared to adults also in flying squirrels^43^, but perhaps the overwinter survival of juveniles is mainly determined by their ability to locate suitable nesting sites^17^. This would explain the current result; that is, if a juvenile is able to locate a suitable nest site, then its response to other environmental variables (food availability and predation risk) appears similar to that of adults. Our data cannot be used to evaluate the exact level of juvenile survival, but in earlier studies on flying squirrels, the mean juvenile survival during the first year has been approximately 15–20% (from June to June^44^). Our apparent overwinter survival for autumn-only boxes was higher (40%; from October to April), but remained clearly lower than overwinter survival in nest boxes used by residents. Our data include cases where we have not observed individuals present in the area (see “Materials and methods”). For example, individuals not detected in spring or autumn but that have occupied the nest box during winter (juveniles or adults) affect the values presented in Fig. 3. That is, the overwinter survival is about 20% in boxes used only in spring, which is clearly smaller than for other nest-box-use categories and is explained by the occupancies not detected in our survey. There is, however, no reason to expect this to affect the current conclusions, because we are not interested on exact survival estimates but patterns driving changes in occupancy levels during different seasons.

The apparent Ural owl predation had a clear negative effect on nest-box occupancy of the flying squirrel, but again against the prediction, the effect was not clear in overwinter survival of apparent juveniles. This unexpected result might indicate that it takes some time from Ural owls to detect the territories occupied by flying squirrels, and, thus, the predatory impact on recently colonised territories remains low. It is clear that a juvenile life before locating a territory is risky (e.g.^44^), but our results indicate that risk of predation is not necessarily higher for juveniles during first winter than it is for adults. For apparent adult flying squirrels, the predation pressure imposed by Ural owls was, however, clear both in winter and in summer. During summer, prey consumption of Ural owls is high due to the feeding of offspring and the Ural owl juveniles are present near the nest site for the whole summer, approximately four months during the post-fledging period. Instead, in the dispersal model (first model in Table 2), the Ural owl presence appeared to have positive effect. That is, nest boxes occupied in spring and oversummer (residents) had lower Ural owl index compared to boxes occupied only in autumn (dispersers). This also is likely explained by the fact that after the arrival of dispersers, predators have had only a short time period to affect site occupancy, compared to the effect on resident individuals during the whole spring and summer.

Temporal occupancy patterns showed two interesting trends: nest-box occupancy from spring to autumn increased, but overwinter survival decreased during our study period (2002–2018). The mechanisms behind these patterns remain unclear, and during the study period the number of flying squirrels using nest boxes has generally increased within the study area. There was no clear temporal trend in catkin availability^36^ and, although, winters warmed during our study period^36^, winter weather had no effect on overwinter survival. Instead, there was a trend of decreasing summer temperature during our study (see “Materials and methods”), and summer temperature was observed to correlate positively with overwinter survival. This relationship could be behind the observed decrease in apparent overwinter survival.

For climate change research, it is central to understand whether the direct effects of weather or biotic interactions determine responses to climate change in different taxa (e.g.^45,46^). Our results suggest the central importance of biotic interactions, in our case the interactions between a boreal rodent, its winter food plants, and main predators. In particular, the role of food plants may be a general pattern in forest ecosystems for species dependent on seed and fruit production of trees. In other words, the effect of climate change may be mediated through the responses of tree mast to changing weather^47,48^, and through the ability of species to respond to these changes^9^. Therefore, biotic interactions determine the species’ response to climate change to a large extent, although ultimately, weather determines this interaction in the case of the flying squirrel and catkin mast.

## Material and methods

### Study area and nest-box occupancy

The study area is located in the Kauhava region, western Finland (62° 54′–63° 16′ N, 22° 54′–23°47′ E; ca. 1,300 km^2^ area; altitude 42 m), where the landscape is mainly characterized by a mosaic of commercially managed coniferous forests, agricultural land and peatland bogs^23,49^. Some mixed and old-growth forests as well as many clear-cuts and sapling areas are also found within the area. The area is sparsely populated, and settlement mainly consists of one-family houses and farmhouses.

The flying squirrel is dependent on natural cavities, which have become scarce in Finnish managed forests, including our study area^40^. In the study area, flying squirrels used nest boxes built for Pygmy owls (*Glaucidium passerinum*) that are set up for research purposes (e.g.^23,40,50^). This nest-box type resembles cavities made by the great spotted woodpecker (*Dendrocopos major*) with the thickness of the front wall > 50 mm and the diameter of the entrance-hole of 45 mm. Nest boxes were grouped so that there are 2 boxes 80–100 m apart within a forest site, the sites being at least 0.8–1.0 km apart^23,40^. The 2 boxes per site were within an average flying squirrel female territory (8 ha^51^), and the data for these boxes were combined, that is, if one of the boxes was occupied the site was classified as occupied. In other words, the site was used as a sampling unit (on average 364 ± 121 nest boxes in 208 ± 61 sites yearly).

The occupancy of the nest boxes by flying squirrels was checked every spring and autumn in 2002–2018. Sites were often visited more than once in both spring and autumn, and we control for the number of visits in our analysis (on average 4 ± 1 visits per year on a site). Occupancy was determined by the presence of flying squirrel nesting material within the nest box (ball-shaped nest made of lichen, moss and other soft material, distinct from the nests built by any other animal in the area). If the nest was not used, the nest material was lacking or was a flat layer in the bottom of the nest box, often covered with bird nest materials. The flying squirrel occupied about 9% of the available nest-boxes^23^. The density of nest boxes was low (0.3 boxes per 1 km^2^) suggesting that nest boxes had only a minor role in the spatial distribution of the flying squirrel population within the area. The nest boxes were in various forest types, but the detection probability in different forest types does not differ substantially in our data^23^.

The occupancy patterns were expected to reflect the seasonal mortality and dispersal patterns described in the introduction of this study (seasonal models: (i) dispersal model, (ii) summer survival model, and (iii) winter survival model). Individuals do have more than one nest during year in nest-boxes, dreys and natural cavities^21^. We could not observe individuals if they did not use nest boxes. Natural cavities were, however, rare near the nest boxes^40^ and the nest boxes were made to resemble natural cavities by using the trunk of spruce (*Picea abies*) or aspen (*Populus tremula*). Communal nesting behavior or reproductive success do not differ for flying squirrels living in these nest boxes and natural cavities in Finland^21^. The lack of cavities means that flying squirrels present in the area had a reason to build nests to nest boxes, because cavities or nest boxes are preferred nesting places over dreys^21^. Thus, there should not be much individuals not using our nest boxes, although it is clear that such a cases do occur (see “Discussion”). The data includes, for example, cases where the residents died during summer, but we did not detect them, because dispersers recolonised the nest box. In practise, the number of such cases remains low in our data. It would simultaneously require that in an occupied nest box (occupancy rate of available nest boxes was on average 9%) the resident adult dies during summer (adult summer mortality is not high) and a disperser arrives to the site, which likelihood for a specific nest box remains low. Finally, we are unaware of species that might prevent flying squirrels from using the nest boxes, except for the Pygmy owl. In spring, 5 to 10% of nest-box sites (3–6% of nest boxes) were occupied by breeding pygmy owls and in autumn 17% of nest-boxes included food-stores of Pygmy owls^50^. Pygmy owls do not prey on flying squirrels but may affect the availability of nest boxes. However, one nest box per forest site was available for flying squirrels even in the sites used by a Pygmy owl, thanks to the study design of two nest boxes per site.

### Winter food

Birch catkins are the main food for the flying squirrel in winter^21^, likely, because the birch is the most abundant deciduous tree in Finnish forests. However, alder catkins are preferred over birch catkins^21^, and recent studies indicate that the availability of alder catkins in the winter and spring preceding reproduction is an important determinant of breeding success^22,25^. Temperature in summer determines catkin production^30^, that is, catkins mature during summer, are available for flying squirrels starting in autumn and stay dormant over winter. Thus, in the current analyses temperature measured in summer is related to next winters’ catkin availability. Catkins flower in spring but flying squirrels may extend the period of catkin usage by storing them^21^.

For birch catkin availability, we used estimates from an annual birch catkin survey conducted by the Natural Resources Institute Finland (www.luke.fi). These data are collected to describe nation-wide pollen conditions in Finland. Catkin production of deciduous trees is spatially auto-correlated at scales of up to a few hundred kilometres in Finland^30,51^, and we used the estimate for central-western Finland, where our study area is located. The birch catkin data for central-western Finland is collected annually at approximately six different locations from 304 trees within the region. We did not have an estimate for alder catkin production, but following earlier studies^22,25,26,52^, we used aerial pollen estimates for central-western Finland as a proxy for alder catkin production (https://www.norkko.fi/). Pollen data were collected by the aerobiology unit of the University of Turku from 10 locations in Finland using EU standard methods and Burkard samplers. The data consisted of accumulated sums of average daily counts of airborne pollen in 1 m^3^ of air during spring^30^. Thus, winter food data used in this study describes yearly changes in catkin availability in the region.

### Weather data

We used mean monthly weather information from the weather station maintained by the Finnish Meteorological Institute in Kauhava^53^. The weather recording station was in the middle of the study area and at the same altitude as the rest of the area. There is minimal spatial variation in mean monthly weather measures within our flat study area. We counted mean temperature and precipitation from monthly means for the following periods: winter (December–February), spring (April–May), summer (June–August) and autumn (October–November). March and September were excluded, as they could not be unequivocally assigned to a specific season and, thus, to life stages of flying squirrels (spring: reproduction; summer: rising juveniles; autumn: dispersal period; winter: surviving from the elements). Including these months to analysis did not change the current results or conclusions.

During the study period of 2002–2018, the temperature had an increasing trend in winter and autumn, and a negative trend in summer (effect of continuous variable year on temperature: in winter positive relationship r^2^ = 0.09; in spring positive r^2^ = 0.01; in summer negative r^2^ = 0.09; in autumn positive r^2^ = 0.1). For precipitation, the trends were positive or non-existing (effect of year on precipitation: in winter positive r^2^ = 0.04, in spring positive r^2^ = 0.02, in summer positive r^2^ = 0.07, in autumn r^2^ = 0).

### Predation pressure

Flying squirrels are negatively affected by the presence of the Ural owl in our study area^23^. Other predators play a lesser role without having major impacts on flying squirrels (the goshawk *Accipiter gentilis*^23^), or are not very common in the area (the pine marten *Martes martes* and the eagle owl *Bubo bubo*^48^). The Ural owl prefers mature mixed and spruce-dominated forest^54^, just like the flying squirrel. Data on Ural owls was collected by surveys on natural cavities and nest boxes and by searching for new nest sites annually in 2002–2018. Long-term studies of birds of prey have been carried out in the Kauhava region (e.g.^40,48,49^), so the locations of Ural owl nests are known. The density of Ural owls was approximately 2 pairs per 10 km^2^ (^48^; M. Hänninen & E. Korpimäki, unpublished data).

Using the data for Ural owl nests located during the field surveys, the predator presence at flying squirrel nest-box sites was described by calculating flat-top bivariate Gaussian kernels around each nest (see^23,55^). Following our earlier analysis^23^, we calculated the kernels with a flat top distance of 500 m, SD of 4 and cut off distance of 5 km. The flat-top part represents the area where the impact of the avian predator is strongest, beyond which it declines, following the Gaussian distribution. The height of the kernel (0–1) at flying squirrel nest box was used as a proxy for predation pressure (referred to as Ural owl index). The kernels were calculated using ArcGIS 10.1 software by Esri and R 3.2.5^55^. The Gaussian kernels were used because the location of nests was known, but we do not know the exact hunting area of individuals. The kernels were based, however, on expert knowledge on likely hunting distance of the species^56^. That is, the hunting effort was assumed to be highest close to the bird’s nest and to remain at a high level within a given distance and then decrease symmetrically in all directions when moving further from the nest.

### Habitat data

The areas of different land use classes within a buffer of 200 m were calculated for each nest box in ArcGIS and R. The buffer corresponds roughly to the estimated home-range size of female flying squirrels^50^. Thus, the selected spatial scale captured habitat composition at the level central for reproductive success. Landscape maps were based on SLICE dataset^57^, two forest classifications from 1997 and 2009 (METLA, https://www.maanmittauslaitos.fi/en/opendata), and Landsat images (https://landsat.usgs.gov/), so that yearly changes in forest cover (e.g. clear-cutting of forest) were taken into account. For a detailed description of map processing, see^40^. We compared which forest composition best describes the squirrel presence and selected the one best fitted to the data based on an Akaike Information Criterion (AIC). That is, model combinations with different forest types and age classes were tested and the one with lowest AIC-values was selected to final models being best fitted for the analysis. The habitat best explaining flying squirrel occurrence included all mature and old spruce and mixed coniferous–deciduous forests. Pure pine forests, which are not preferred by the species^21^, were excluded.

The available habitat data ended in the year 2015 because we had no information for changes in the forest cover after 2015. We updated the habitat data until 2018 with the values for 2015, but in the end decided to use only the habitat data until 2015 and omitted it from the final models, because it had no effect (see “Results”). Thus, we gained full power to analyse the effects of weather, winter food and predator pressure on flying squirrel occupancy patterns.

### Analyses—dispersal model, summer survival model, and winter survival model

We built three binary models (using GLIMMIX in SAS 9.4. software) with nest-box occupancy in different seasons as a response variable. In each model, the nest-box site was a repeated factor (using generalized estimation equations, GLIMMIX SAS) and the year and average number of nest-box visits per year were continuous explanatory variables. To simplify the models, we used an AIC comparison to select the weather variables that were best fitted to the model (AIC < 2 were included in final models; interactions between precipitation and temperature were included to the analysis). Similarly, either the alder or birch index was selected for final models based on the smaller AIC value. Consequently, the variables selected for the final models were not strongly correlated (variance inflation factor < 4). To be able to compare model estimates of different explanatory variables, we standardized the continuous variables as (x − μ)/σ, where x is a raw data value, μ is the mean, and σ is the standard deviation.

In the dispersal model (model i, see introduction), nest boxes used only in autumn (coded with value 2; potential natal dispersal cases) were compared to boxes used only in spring or both in spring and summer (coded with value 1). The final model was: value 2 vs value 1 = Alder pollen during the previous winter + the Ural owl index + summer rain + summer temperature + year + number of nest-box visits; site as repeated factor. In other words, the preliminary AIC analysis indicated that summer rain and temperature were the best-fitted weather variables for this model.

In the summer survival model (ii), spring only boxes (value 1) were compared to boxes used in both spring and autumn (oversummer occupancy; value 2). This model describes the apparent summer mortality of resident adults. The final model was: 2 vs 1 = birch catkins in the previous winter + the Ural owl index + summer rain + winter temperature (previous year) + year + number of nest-box visits; site as repeated factor. In this model, interaction term between summer rain and temperature (see predictions in aims) was not significant and dropped from the final model during preliminary AIC analysis of weather variables.

In the overwinter survival model (iii), we analysed occupancy status the following spring (i.e. after the winter; 2 = yes or 1 = no). Interaction terms between occupancy status (autumn only, i.e. dispersers vs spring and autumn, i.e. residents) and food or Ural owl index were included in the model. This was done to compare whether the responses differed between apparent dispersers (autumn only) and resident adults (spring and autumn). The overwinter survival model was run without spring-only boxes (the result was the same if we included spring-only boxes), because these were not expected to be occupied in the next winter. The final model was 2 vs 1 = occupancy status from the previous year (class variable: autumn only 1, both spring and autumn 2; from now on called season) + birch catkins during the winter + the Ural owl index + summer temperature (previous year) + winter temperature (current winter) + winter rain (current winter) + autumn rain (previous autumn) + season*Ural owl index + season*birch catkins + year + number of nest-box visits; site as repeated factor.
